# Food Insecurity Is Associated with Increased Risk of Non-Adherence to Antiretroviral Therapy among HIV-Infected Adults in the Democratic Republic of Congo: A Cross-Sectional Study

**DOI:** 10.1371/journal.pone.0085327

**Published:** 2014-01-15

**Authors:** Patou Masika Musumari, Edwin Wouters, Patrick Kalambayi Kayembe, Modeste Kiumbu Nzita, Samclide Mutindu Mbikayi, S. Pilar Suguimoto, Teeranee Techasrivichien, Bhekumusa Wellington Lukhele, Christina El-saaidi, Peter Piot, Masako Ono-Kihara, Masahiro Kihara

**Affiliations:** 1 Department of Global Health and Socio-epidemiology, Kyoto University School of Public Health, Kyoto, Japan; 2 Department of Sociology, Research Centre for Longitudinal and Life Course Studies, University of Antwerp, Antwerp, Belgium; 3 Kinshasa University School of Public Health, University of Kinshasa, Kinshasa, Democratic Republic of Congo; 4 Centre Hospitalier Monkole, Kinshasa, Democratic Republic of Congo; 5 London School of Hygiene and Tropical Medicine, London, United Kingdom; Fundacion Huesped, Argentina

## Abstract

**Background:**

Food insecurity is increasingly reported as an important barrier of patient adherence to antiretroviral therapy (ART) in both resource-poor and rich settings. However, unlike in resource rich-settings, very few quantitative studies to date have investigated the association of food insecurity with patient adherence to ART in Sub-Saharan Africa. The current study examines the association between food insecurity and adherence to ART among HIV-infected adults in the Democratic Republic of Congo (DRC).

**Methods and Findings:**

This is a cross-sectional quantitative study of patients receiving ART at three private and one public health facilities in Kinshasa, DRC. Participants were consecutively recruited into the study between April and November 2012. Adherence was measured using a combined method coupling pharmacy refill and self-reported adherence. Food insecurity was the primary predictor, and was assessed using the Household Food Insecurity Access Scale (HFIAS). Of the 898 participants recruited into the study, 512 (57%) were food insecure, and 188 (20.9%) were not adherent to ART. Food insecurity was significantly associated with non-adherence to ART (AOR, 2.06; CI, 1.38–3.09). We also found that perceived harmfulness of ART and psychological distress were associated respectively with increased (AOR, 1.95; CI, 1.15–3.32) and decreased (AOR, 0.31; CI, 0.11–0.83) odds of non-adherence to ART.

**Conclusion:**

Food insecurity is prevalent and a significant risk factor for non-adherence to ART among HIV-infected individuals in the DRC. Our findings highlight the urgent need for strategies to improve food access among HIV-infected on ART in order to ensure patient adherence to ART and ultimately the long-term success of HIV treatment in Sub-Saharan Africa.

## Introduction

The benefits of antiretroviral therapy (ART) in reducing HIV/AIDS related-morbidity and mortality are extensively documented [1]–[3]. Recent evidence indicates that early initiation of ART substantially reduces sexual transmission of HIV at individual [4], [5] and population levels [6]; conferring ART a crucial place in both treatment and prevention of HIV/AIDS.

Prior studies have shown that, without high and sustained adherence levels, both therapeutic and public health benefits of ART cannot be secured, and that individuals with sub-optimal levels of adherence have higher risk of incomplete viral suppression, disease progression, and development of drug resistance [7]–[9].

Our understanding of adherence to ART has substantially increased over the past years with a wide documented range of factors influencing ART adherence across differing settings in developed and developing countries [10]–[17]. Recently, food insecurity has emerged as a key structural barrier that affects adherence to ART in both resource-rich and constrained settings. Studies from British Columbia [18], San Francisco [19], Atlanta [20], [21], and France [22] have found lower levels of medication adherence among food insecure individuals on ART. Similarly, a number of, predominantly qualitative, studies from Sub-Saharan Africa (SSA) have documented food insecurity as an important barrier to ART adherence [12], [23]–[26]. Furthermore, food insecurity was shown to be independently related to poor virologic response and mortality even when adjusting for patient adherence to ART [19], [27], [28].

Food insecurity parallels, and is viciously intertwined with the AIDS epidemic in SSA, both having a damaging impact in the region [29]. SSA accounts for 69% of the people infected with HIV worldwide, and is home to 234 million (26.8%) people classified as undernourished [1], [30]. In the Democratic Republic of Congo (DRC), the research setting of the current study, the Multi Indicator Cluster Survey (MICS) revealed that approximately 33% of the households were experiencing food insecurity in 2010, with figures ranging from 5% in the Kinshasa City to nearly 60% in some eastern provinces of the DRC [31]. The national HIV program reports a HIV prevalence of 2.57% among the general population, with only 12.3% of eligible patients having access to ART [32].

A review of the literature revealed that, unlike in resource-rich settings, very few quantitative studies to date, using a validated measure of food insecurity, have documented the association between food insecurity and patient adherence to ART in SSA and the developing world at large [26], [33]. Additionally, literature on patient adherence to ART in the DRC, the second largest country in Africa, remains exceptionally scarce. In our preliminary qualitative study, we found that food insecurity, financial constraints, forgetfulness, and fear of disclosure/stigma were common barriers to ART adherence; while religious beliefs were both a barrier and facilitator of ART adherence among HIV-positive adults in the DRC [23].

The current study, grounded in the results of our qualitative study [23], aims at quantitatively assessing factors associated with adherence and more specifically, documenting the prevalence of food insecurity, and its effect on ART adherence among HIV-positive adults receiving ART in the DRC. Such information is crucial for guiding context-specific interventions to promote patient adherence to ART.

## Methods

### Ethics Statement

This study was granted ethical approval from the Committee for Research on Human Subjects at Kyoto University and the Kinshasa University School of Public Health Ethics Review Committee in the DRC. All the participants provided written informed consents before being interviewed.

### Study Design, Participants and Setting

This is a cross-sectional study conducted in Kinshasa, DRC, between April and November 2012. Participants were consecutively recruited into the study from one public health facility: Hopital Provincial General de Référence de Kinshasa (HGPRK) and three private health institutions which included one treatment site of the NGO Actions Communautaires Sida/Avenir Meilleur pour les Orphelins (ACS/Amo-Congo), three treatment sites of the Armée du salut (Salvation Army), and two treatment sites of the Centre Hospitalier Monkole (CHM). Recruitment sites were in geographically dispersed locations and served patients coming from all the townships of Kinshasa. At the time the study was conducted, around 1,000, 2,900, 2,300, and 1,100 patients were receiving free ART respectively at the HGPRK, ACS/Amo-Congo, Armée du salut, and the CHM, and none of the facilities provided food or any kind of nutrition aid to patients. We collected data using an interviewer-administered questionnaire, which we designed based on the findings from our prior qualitative study [23] supplemented with other relevant questions obtained from the available literature [34]–[38]. The questionnaire was piloted in a sample of 20 respondents (not included in the final sample) to ensure clarity prior to conducting interviews, and it showed an overall good test-retest reliability performed within a one month interval. The interviews were conducted in French or Lingala, the most commonly used languages in Kinshasa, and the interviewers were provided guidance on questionnaire administration over two training sessions organized by the research team. Participants were included in the study provided that they were at least 18 years old, on ART for at least 6 months, and had given written informed consent. Participants were compensated for their time and transportation with an amount of 3 US dollars.

### Measures

#### Primary outcome

The primary outcome of interest was adherence to ART, and was assessed using a composite measure coupling both pharmacy refill and self-reported adherence. Pharmacy refill adherence measures were shown to be reliable in assessing adherence, and to correlate well with virological and clinical outcomes in resource limited settings [39]–[42], and the combination with self-reported adherence performed better in predicting virological failure than other singular methods [43].

The pharmacy refill adherence in this study is based on a variation of the medication possession ratio (MPR), a measure of the proportion of days a patient has his/her medication on hand, and was calculated by dividing the number of days late for pharmacy refills by the total days on ART, and then subtracting this proportion from 100% [40], [44]. We calculated the average pharmacy refill rate of adherence for each patient over the preceding six months. When applicable, the number of days being late for pharmacy refills was adjusted to account for cases where patients were provided with more medication than needed. Patients with pharmacy adherence levels <95% were categorized as non-adherent to ART [8].

To measure self-reported adherence, we adopted a validated tool that assesses adherence over the previous seven days [34]. The tool contains questions that first measure adherence over shorter time frames (yesterday and the day before yesterday), and an aided-recall question for situations that can potentially lead to missed ART doses with the intent to facilitate a more accurate reporting of the number of pills skipped during the previous seven days, and to limit the influence of forgetfulness. Self-reported non-adherence was defined as taking <95% of the prescribed pills over the previous seven days [8].

In this study, participants were categorized as non-adherent to ART when they were non-adherent to either one or both of the measures of adherence described above; otherwise, they were considered to be adherent.

#### Primary independent variable

Food insecurity was the primary independent variable of this study, and was measured by the Household Food Insecurity Access Scale (HFIAS) [35]. The HFIAS is a validated instrument and has been shown to distinguish food insecure from food secure households across different cultural contexts. It is a set of nine questions designed to reflect universal domains of the experience of food insecurity including 1) anxiety and uncertainty about the household food supply, 2) insufficient quality (includes variety and preferences of the type of food), and 3) insufficient food intake and its physical consequences. We presented results in a categorical format including 1) food secure, 2) mildly food insecure 3) moderately food insecure, and 4) severely food insecure, which we dichotomized into food insecure versus food secure. The Cronbach’s alpha was 0.97, demonstrating a high internal consistency of the scale in our sample.

#### Other covariates

***Internalized AIDS stigma***: The Internalized AIDS-Related Stigma Scale was used to assess the internalized AIDS stigma [36]. The items were administered on a 5-point Likert-scale ranging from strongly disagree to strongly agree. Strongly disagree, disagree, and neutral were converted to 0 and agree and strongly agree to 1. Scale score ranged 0–6; participants who obtained relatively high scores on the scale (>2) were compared to those who had low scores (≤2) [45]. The scale showed a moderate degree of internal consistency with Cronbach’s alpha = 0.60.

***Psychological distress (Depression, anxiety disorders)***: Mental health status was assessed using the Kessler-6 scale, a standardized and validated screening tool for non-specific psychological distress including depression and anxiety disorders [37]. Participants were asked on a 5-points Likert scale ranging from 0 (none of the time) to 4 (all of the time) how often they felt 1) nervous, 2) hopeless, 3) restless or fidgety, 4) so depressed that nothing could cheer you up, 5) that everything was an effort, and 6) worthless. Scores equal to or higher than 13 indicate higher probability of psychological distress. There was a high internal consistency of the scale in our sample with Cronbach’s alpha = 0.89.

***Other variables***: other covariates included the household wealth index [46], socio-demographic characteristics (10 items), HIV/AIDS disease-related variables (4 items) and HIV/AIDS knowledge (8 items), ART-related variables (8 items), perceptions about HIV/AIDS and ART (12 items), alcohol and drug use (2 items), social support [47], and perceived quality of health care (6 items) [38]. (See Text S1 for additional information on variables).

### Statistical Analysis

Data was analyzed using SPSS (PASW) for Windows 17.0 (SPSS Inc., Chicago, Illinoi, USA). Univariate analysis was conducted to obtain descriptive statistics of all the variables. Bivariate analyses were performed using Chi-square tests for categorical variables and Mann Whitney U-test for continuous variables. We included in the analysis nonresponse cases on items related to perceptions about HIV/AIDS and ART; their exclusion did not affect the results of our analysis (See Table S1, which shows frequency of nonresponse for perception items). We grouped nonresponses with participants whose answers were “disagree” and “don’t know” since they were similar with respect to their odds ratios when compared to participants who agreed to the assertions. Factors associated with non-adherence by bivariate analysis with P value ≤0.10 and those considered epidemiologically important were entered into a multivariate logistic regression model to obtain adjusted odds ratios (AOR) and 95% confidence intervals (CI). “Frequency of ART” and “regimen drugs” were not included in the multivariate model, even though both variables had each a category with P value ≤0.10, overall the two variables did not meet the inclusion criteria, and we excluded “duration of HIV infection” from the model because of its multicollinearity with “duration of ART”.

## Results

### Participant Characteristics

A total of 898 participants completed the study and 25 declined participation, giving a response rate of 97.3%. The median age was 44 years [Interquartile range (IQR): 38–51]. The majority of participants were female (72.2%), without standard employment (76.4%), had completed at least secondary school (75.3%), and Christians (87.9%) by religion (Table 1). The median treatment duration was 41 months (IQR: 18–64); most participants were on first line regimens (97.9%), and on a twice a day dosing schedule (92.3%). (Table 2).

**Table 1:** Sociodemographic characteristics of non-adherent and adherent participants on ART recruited in Kinshasa, DRC.

	Non-Adherent (n = 188)	Adherent (n = 710)	Total (n = 898)	Crude OR (95% CI)	P value
	n (%)	n (%)	n (%)		
**Gender**					
Male	49 (26.1)	201 (28.3)	250 (27.8)	1.00	
Female	139 (73.9)	509 (71.7)	648 (72.2)	1.12 (0.77–1.61)	0.603
**Educational level**					
Primary school or less	46 (24.5)	176 (24.8)	222 (24.7)	1.00	
Secondary school	110 (58.5)	420 (59.2)	530 (59.0)	1.00 (0.68–1.47)	1.000
University	32 (17.0)	114 (16.0)	146 (16.3)	1.07 (0.64–1.78)	0.885
**Marital status**					
Married/cohabitating	63 (33.5)	261 (36.8)	324 (36.1)	1.00	
Single	43 (22.9)	164 (23.1)	207 (23.1)	1.08 (0.70–1.67)	0.793
Divorced/Separated	29 (15.4)	83 (11.7)	112 (12.5)	1.44 (0.87–2.39)	0.191
Widowed	53 (28.2)	202 (28.4)	255 (28.4)	1.08 (0.72–1.63)	0.768
**Religion**					
Catholic Christian	54 (28.7)	194 (27.3)	248 (27.6)	1.00	
Protestant Christian	22 (11.7)	125 (17.6)	147 (16.4)	0.63 (0.36–1.09)	0.127
Revival churches Christian	85 (45.2)	309 (43.5)	394 (43.9)	0.98 (0.67–1.45)	1.000
Others*	27 (14.4)	82 (11.5)	109 (12.1)	1.18 (0.69–2.00)	0.627
**Employment status**					
Employed	37 (19.7)	175 (24.6)	212 (23.6)	1.00	
Unemployed	151 (80.3)	535 (75.4)	686 (76.4)	1.33 (0.89–1.98)	0.184
**IGA**					
Yes	136 (72.3)	478 (67.3)	614 (68.4)	1.00	
No	52 (27.7)	232 (32.7)	284 (31.6)	0.78 (0.55–1.12)	0.220
	Median (IQR)	Median (IQR)	Median (IQR)		
**Age (years)**	43 (36. 25–50)	44 (38–51)	44 (38–51)		0.150
**Household size**	5 (4–7)	5 (3–7)	5 (3–7)		0.426
**Financial dependents** †	3 (1.25–5)	3 (1–5)	3 (1–5)		0.820
**Household wealth index**	0.08 (−2.29 to 2.55)	0.16 (−2.44 to 2.42)	0.16 (−2.43 to 2.42)		0.823

Other: Muslim, Kimbaguist, None;

number of financial dependents; ART, antiretroviral therapy; IGA, income generating activity; OR, odds ratio; IQR, inter-quartile range; DRC, Democratic Republic of Congo.

**Table 2:** Bivariate analysis of factors associated with Non-adherence to ART.

	Non-Adherent (n = 188)	Adherent (n = 710)	Total (n = 898)	Crude OR (95% CI)	p value
	n (%)	n (%)	n (%)		
**Food insecurity**					
No	53 (28.2)	333 (46.9)	386 (43.0)	1.00	
Yes	135 (71.8)	377 (53.1)	512 (57.0)	2.25 (1.58–3.19)	0.000
**Disclosure**					
No	36 (19.1)	170 (24.0)	206 (22.9)	1.00	
Yes	152 (80.9)	540 (76.0)	692 (77.1)	1.32 (0.88–1.98)	0.196
**Psychological distress**					
No (Score:0–12)	183 (97.3)	663 (93.4)	846 (94.2)	1.00	
Yes (Score:13–24)	5 (2.7)	47 (6.6)	52 (5.8)	0.38 (0.15–0.98)	0.039
**Internalized stigma**					
Score:0–2	123 (65.4)	483 (68.0)	606 (67.5)	1.00	
Score:3–6	65 (34.6)	227 (32.0)	292 (32.5)	1.12 (0.80–1.57)	0.555
**Social support from family**					
No	96 (51.1)	417 (58.7)	513 (57.1)	1.00	
Yes	92 (48.9)	293 (41.3)	385 (42.9)	1.36 (0.98–1.88)	0.071
**Social support from non-family members**					
No	137 (72.9)	544 (76.6)	681 (75.8)	1.00	
Yes	51 (27.1)	166 (23.4)	217 (24.2)	1.22 (0.84–1.75)	0.331
**Opportunistic infection**					
No	181 (96.3)	683 (96.2)	864 (96.2)	1.00	
Yes	7 (3.7)	27 (3.8)	34 (3.8)	0.97 (0.41–2.28)	1.000
**HIV-infected individual(s) in the household**					
No	160 (85.1)	554 (78.0)	714 (79.5)	1.00	
Yes	28 (14.9)	156 (22.0)	184 (20.5)	0.62 (0.40–0.96)	0.042
**Alcohol intake** *					
No	139 (73.9)	585 (82.4)	724 (80.6)	1.00	
Yes	49 (26.1)	125 (17.6)	174 (19.4)	1.65 (1.13–2.40)	0.012
**Tobacco smoking**					
No	179 (95.2)	680 (95.8)	859 (95.7)	1.00	
Yes	9 (4.8)	30 (4.2)	39 (4.3)	1.14 (0.53–2.44)	0.893
**Knowledge of HIV/AIDS**					
Good (≥5)	164 (87.2)	622 (87.6)	786 (87.5)	1.00	
Poor (≤4)	24 (12.8)	88 (12.4)	112 (12.5)	1.03 (0.63–1.67)	0.990
**Regimen**					
1st line	184 (97.9)	695 (97.9)	879 (97.9)	1.00	
2nd line	4 (2.1)	15 (2.1)	19 (2.1)	1.00 (0.33–3.07)	1.000
**Regimen drugs**					
3TC+TDF+EFV or NVP	10 (5.3)	67 (9.4)	77 (8.6)	1.00	
3TC+AZT+EFV or NVP or LPV/rit	165 (87.8)	575 (81.0)	740 (82.4)	1.92 (0.96–3.82)	0.080
3TC+D4T+EFV or NVP	9 (4.8)	53 (7.5)	62 (6.9)	1.13 (0.43–3.00)	0.990
ABC+DDI+LPV/r	4 (2.1)	15 (2.1)	19 (2.1)	1.78 (0.49–6.47)	0.597
**Pill burden**					
1–4	169 (89.9)	650 (91.5)	819 (91.2)	1.00	
≥5	19 (10.1)	60 (8.5)	79 (8.8)	1.21 (0.70–2.09)	0.570
**Frequency of ART**					
Once/day	5 (2.7)	45 (6.3)	50 (5.6)	1.00	
Twice/day	179 (95.2)	650 (91.5)	829 (92.3)	2.47 (0.96–6.33)	0.075
Thrice/day	4 (2.1)	15 (2.1)	19 (2.1)	2.40 (0.56–10.11)	0.247
**Refill Schedule**					
Every 2 or 3 months	43 (22.9)	204 (28.7)	247 (27.5)	1.00	
Every month	145 (77.1)	506 (71.3)	651 (72.5)	1.35 (0.93–1.98)	0.132
**Duration of HIV infection**					
<48 months	76 (40.4)	342 (48.2)	418 (46.5)	1.00	
≥48 months	112 (59.6)	368 (51.8)	480 (53.5)	1.37 (0.98–1.89)	0.070
Median (IQR)	48 (24–72)	48 (24–72)	48 (24–72)		
**Duration of ART**					
<48 months	94 (50.0)	421 (59.3)	515 (57.3)	1.00	
≥48 months	94 (50.0)	289 (40.7)	383 (42.7)	1.45 (1.05–2.01)	0.027
Median (IQR)	48.5(24–72)	40 (18–62)	41 (18–64)		
**Perceived quality of care**					
Not satisfied (0–2)	10 (5.3)	23 (3.2)	33 (3.7)	1.00	
Moderately satisfied (3–4)	22 (11.7)	86 (12.1)	108 (12.0)	0.58 (0.24–1.41)	0.340
Highly satisfied (5–6)	156 (83.0)	601 (84.6)	757 (84.3)	0.59 (0.27–1.28)	0.263
**Treatment sites**					
HPGRK	13 (6.9)	86 (12.1)	99 (11.0)	1.00	
ACS/AMO CONGO	94 (50.0)	208 (29.3)	302 (33.6)	2.99 (1.58–5.62)	0.001
MONKOLE	35 (18.6)	165 (23.2)	200 (22.3)	1.40 (0.70–2.79)	0.423
ARMEE DU SALUT	46 (24.5)	251 (35.4)	297 (33.1)	1.21 (0.62–2.35)	0.684
**Travel time (in hours)** †					
<1	103 (54.8)	398 (56.1)	501 (55.8)	1.00	
1–<2	66 (35.1)	251 (35.3)	317 (35.3)	1.01 (0.71–1.43)	0.999
≥2	19 (10.1)	61 (8.6)	80 (8.9)	1.20 (0.68–2.10)	0.615

Defined as alcohol consumption at least once a month;

travel time from home to health facility expressed in hour(s); ART, antiretroviral therapy; OR, odds ratio; CI, confidence interval; 3TC = Lamivudine; TDF = Tenofovir; EFV = Efavirenz; NVP = Nevirapine; AZT = Zidovudine; LPV/r = Lopinavir/Ritonavir, D4T = Stavudine, ABC = Abacavir; DDI = Didanosine; IQR, inter-quartile range; HPGRK, Hopital Provincial General de Référence de Kinshasa; ACS/AMO Congo, Actions Communautaires Sida/Avenir meilleur pour les orphelins.

### Food Insecurity and Adherence Assessment

Based on the HFIAS, 386 participants (43.0%) were classified as food secure, 9 (1.0%) as mildly food insecure, 46 (5.1%) as moderately food insecure and 457 (50.9%) as severely food insecure (See Table S2, which shows details of participants’ food security status). The overall prevalence of food insecurity among our participants was 57% (Table 2). 188 (20.9%) participants were categorized as non-adherent to ART in respect to the definition of adherence described above (See Table S3, which shows details of participants’ adherence status).

### Bivariate Associations between Independent Variables and ART Adherence

Factors significantly associated with non-adherence in the bivariate analysis included food insecurity [odds ratio (OR), 2.25; CI, 1.58–3.19; *P* = 0.000)], psychological distress (OR, 0.38; CI, 0.15–0.98; *P* = 0.039), presence of (an)other HIV-infected individual(s) in the household (OR, 0.62; CI, 0.40–0.96; *P* = 0.042), alcohol intake (OR, 1.65; CI, 1.13–2.40; *P* = 0.012), duration of ART [≥48 months] (OR, 1.45; CI, 1.05–2.01; *P* = 0.027), perceived ART harmfulness (OR, 1.68; CI, 1.10–2.55; *P* = 0.042), and the beliefs that God or prayers could cure HIV (OR, 2.10; CI, 1.45–3.04; *P* = 0.000), or that ART worked better when associated with prayers (OR, 1.73; CI, 1.23–2.43; *P* = 0.002), and receiving treatment from ACS/Amo-Congo (OR, 2.99; CI, 1.58–5.62; *P* = 0.001). (Table 2 & Table 3).

**Table 3:** Perceptions about HIV/AIDS and ART.

	Non-Adherent (n = 188)	Adherent (n = 710)	Total (n = 898)	Crude OR (95% CI)	P value
	n (%)	n (%)	n (%)		
***Sociocultural/Religious beliefs***					
**God/prayer can cure HIV**					
Agree	143 (76.1)	427 (60.1)	570 (63.5)	2.10 (1.45–3.04)	0.000
Else	45 (23.9)	283 (39.1)	328 (36.5)	1.00	
**Traditional healers/medicine can cure HIV**					
Agree	9 (4.8)	35 (4.9)	44 (4.9)	0.97 (0.45–2.05)	1.000
Else	179 (95.2)	675 (95.1)	854 (95.1)	1.00	
**ART works better when combined with prayers**					
Agree	126 (67.0)	383 (53.9)	509 (56.7)	1.73 (1.23–2.43)	0.002
Else	62 (33.0)	327 (46.1)	389 (43.3)	1.00	
***Perceptions about ART and food***					
**ART is not necessary without food**					
Agree	37 (19.7)	154 (21.7)	191 (21.3)	0.88 (0.59–1.32)	0.618
Else	151 (80.3)	556 (78.3)	707 (78.7)	1.00	
**ART is not effective without food**					
Agree	20 (10.6)	77 (10.8)	97 (10.8)	0.97 (0.58–1.64)	1.000
Else	168 (89.4)	633 (89.2)	801 (89.2)	1.00	
**ART can be harmful without food**					
Agree	58 (30.9)	220 (31.0)	278 (31.0)	0.99 (0.79–1.40)	1.000
Else	130 (69.1)	490 (69.0)	620 (69.0)	1.00	
***Perceptions about ART adherence***					
**Short treatment interruption is not harmful** **to a long-term ART user**					
Agree	10 (5.3)	71 (10.0)	81 (9.0)	0.50 (0.25–1.00)	0.064
Else	178 (94.7)	639 (90.0)	817 (91.0)	1.00	
**Skipping few ART doses is not harmful** **to a long-term ART user**					
Agree	10 (5.3)	48 (6.8)	58 (6.5)	0.77 (0.38–1.56)	0.584
Else	178 (94.7)	662 (93.2)	840 (93.5)	1.00	
**Skipping ART doses can worsen the disease**					
Agree	135 (71.8)	434 (61.1)	569 (63.4)	0.69 (0.46–1.03)	0.087
Else	53 (28.2)	276 (38.9)	329 (36.6)	1.00	
**ART should be taken life-long**					
Agree	172 (91.5)	651 (91.7)	823 (91.6)	1.00 (0.45–2.23)	1.000
Else	16 (8.5)	59 (8.3)	75 (8.4)	1.00	
***Perceptions about ART***					
**Perceived effectiveness of ART**					
Yes	176 (93.6)	630 (88.7)	806 (89.8)	1.86 (0.99–3.49)	0.067
No	12 (6.4)	80 (11.3)	92 (10.2)	1.00	
**Perceived ART harmfulness**					
Yes	29 (15.4)	70 (9.9)	99 (11.0)	1.68 (1.10–2.55)	0.042
No	159 (84.6)	640 (90.1)	799 (89.0)	1.00	

ART, antiretroviral therapy; OR, odds ratio; CI, confidence interval.

Table 4 shows reasons reported by participants for skipping ART doses during the previous seven days. 36 (39.5%) participants cited forgetfulness, 17 (18.6%) were unable to pay for the medical consultation or for transport, 13 (14.2%) ran out of pills, 11 (12.0%) reported lack of food, 7 (7.6%) had travelled, and other reasons were reported in much lower proportions.

**Table 4:** Reasons for missing ART doses during the previous seven days.

Reason	Frequency (n = 91)	%
Forgetfulness	36	39.5
Unable to pay for transport/medical consultation	17	18.6
Ran out of pills	13	14.2
Lack of food	11	12
Travel	7	7.6
Pill fatigue	3	3.2
Alcohol	2	2.1
Away from home	2	2.1
Side effects	1	1.1
Felt tired	1	1.1
Felt depressed	1	1.1
Fell asleep	1	1.1

ART, antiretroviral therapy; Multiple responses are possible.

### Multivariate Analysis

In the multivariate analysis (Table 5), food insecurity was strongly associated with non-adherence. Food insecure participants were two times more likely to be non-adherent to ART compared to those who were food secure (AOR, 1.99; CI, 1.36–2.90, *P* = 0.000). Other factors significantly associated with non-adherence to ART included alcohol intake (AOR, 1.55; CI, 1.02–2.34, *P* = 0.037), and perceived ART harmfulness (AOR, 2.06; CI, 1.30–3.27, *P* = 0.002). Paradoxically, we found that participants who had psychological distress (depression, anxiety) as measured by the K6-scale, had lower odds of non-adherence (AOR, 0.34; CI, 0.12–0.90, *P* = 0.030), as well as those who reported that skipping ART doses could worsen the disease (AOR, 0.58; CI, 0.38–0.88, *P* = 0.012).

**Table 5:** Multivariate analysis of factors associated with non-adherence to ART.

	Adjusted OR	95% CI	P value
Food insecurity yes (vs no)	1.99	1.36–2.90	0.000
Alcohol intake yes (vs no)	1.55	1.02–2.34	0.037
Internalized stigma score:3–6 (vs score:0–2)	1.11	0.76–1.61	0.571
Social support from family yes (vs no)	1.26	0.90–1.78	0.174
Psychological distress yes (vs no)	0.34	0.12–0.90	0.030
Duration of ART ≥48 months (vs <48 months)	1.27	0.90–1.80	0.170
Perceived ART harmfulness yes (vs no)	2.06	1.30–3.27	0.002
Perceived effectiveness of ART yes (vs no)	1.19	0.16–8.93	0.859
Short treatment interruption is not harmful to a long-term ART user Agree (vs else)	0.55	0.27–1.14	0.110
Skipping ART doses can worsen the disease Agree (vs else)	0.58	0.38–0.88	0.012
God/prayer can cure HIV/AIDS Agree (vs else)	1.48	0.92–2.37	0.098
ART works better when combined with prayer Agree (vs else)	1.01	0.64–1.59	0.952
HIV-infected person(s) in the household Yes (vs no)	0.72	0.45–1.15	0.173
Treatment sites			
HPGRK	1.00		
ACS/AMO CONGO	1.87	0.26–13.34	0.531
MONKOLE	0.98	0.13–7.07	0.988
ARMEE DU SALUT	0.77	0.10–5.47	0.798

ART, antiretroviral therapy; OR, odds ratio; CI, confidence interval; HPGRK, Hopital Provincial General de Référence de Kinshasa; ACS/AMO CONGO, Actions Communautaires Sida/Avenir Meilleur pour les Orphelins.

## Discussion

Designed based on the findings of our preceding qualitative study that food insecurity is a prominent structural barrier to ART adherence [23], this study is one of the first to quantitatively demonstrate the association of food insecurity with ART nonadherence in a Sub-Saharan African country. Our findings corroborate previous qualitative studies from SSA [12], [23]–[25], quantitative studies in resource rich settings [18]–[22], and recent findings from a longitudinal cohort study in rural Uganda [26].

Because of the cross-sectional nature of this study, interpretation of the results could be multiple. Firstly the observed association could be confounded by a third factor that was associated with both ART non-adherence and food insecurity. Secondly, we could assume that ART non-adherence was causal to food insecurity. Lastly, food insecurity could be causal to ART non-adherence.

The first possibility could be the case with poverty as a most likely confounder since prior studies have shown that trade-offs between subsistence needs and health care needs resulted in participants missing clinic visits or giving up ART for food in financially constrained individuals [24], [48]. However, our results failed to support this possibility because household wealth index and employment status were not associated with ART non-adherence in bivariate analyses and our multivariate analysis was adjusted for these variables. Residual confounding of poverty due to insufficient sensitivities of these measures to rate poverty seemed unlikely because both variables were strongly associated with food insecurity (*P*<0.001). The second possibility seemed also unlikely because such a narrative had never been documented in qualitative studies including our study [12], [23]–[25] and because ART non-adherence was not associated with any financial measures such as household wealth index and employment status. The third possibility is therefore most likely the case. This is strongly supported by the recent findings from a study in rural Uganda showing that food insecurity is longitudinally associated with non-adherence to ART [26] and preliminary experimental studies that showed that food supplementation improved the adherence to ART among food-insecure adults in Zambia [49], [50].

However, possible mechanisms through which food insecurity could lead to ART non-adherence remained unclear. Our qualitative study identified two perceptions held by the participants as potential mechanisms including ART can be harmful or is not effective when taken without food [23]. In this study, we tested these hypotheses including the questions on participants’ perception on the harmfulness or effectiveness of ART when taken without food but failed to show any association of these perceptions with ART non-adherence. Possible mechanisms may include forgetfulness. Although our study was not designed to compare participants’ forgetfulness between the non-adherent and adherent groups as it was asked only as a branch question to the participants who reported ART non-adherence, forgetfulness was cited as one of the most frequent reasons for skipping ART doses in this study. In a qualitative study in Uganda, participants reported forgetting their daily ART doses as a result of spending most of their time working to obtain food [24], suggesting that food insecure individuals may particularly be prone to forgetfulness. In future studies, this hypothesis should be tested by introducing the question on general forgetfulness on daily ART doses for both groups as well as the question on food insecurity and creating an interaction term between the two.

Whatever the exact mechanism, however, the implications of the results of this study could be far-reaching, considering the vast number of people suffering from food insecurity and the estimated 6 million of people living with HIV on ART treatment in SSA [1], [31], and the fact that food insecurity disproportionately affects HIV-infected individuals [51]–[54]. In our study, over half (57%) of our participants were food insecure and alarmingly, most of them were severely insecure, a rate much higher than that previously reported in the general population in Kinshasa City [32]. In view of the threat of food insecurity to the long-term success of HIV/AIDS treatment programs in SSA, which hosts the largest number of people on and in need of ART, with a very restricted access to second or third line therapy [1], it is crucial to integrate food security strategies into HIV treatment programs [55], [56]. Though, clinic-based short-term intervention studies in SSA have shown promising results for improving patient adherence to ART using food assistance [49], [50]; in order to be sustainable over time, such intervention should be built on a clear understanding of context-specific determinants of food insecurity, and packaged into a holistic approach that takes into account local socio-cultural and structural correlates of ART adherence.

Besides food insecurity our study identified a number of factors associated with ART non-adherence. Consistent with prior studies [57], [58], participants who perceived that ART was harmful had higher odds of non-adherence. Most of them believed so because of ART-related side effects, or the fact that ART was a life-long medication. We observed an association between alcohol use and non-adherence to ART. Participants who reported consuming alcohol at least once a month had a 50% increased odds of being non-adherent to ART. This is consistent with previous studies showing that alcohol use negatively affected patient adherence even for moderate levels of consumption [59], [60]. Our findings also indicated that participants who were on ART for more than 4 years were more likely to be non-adherent compared to those who were placed on ART since less than 4 years. Although this was only significant in bivariate analysis, it still supports previous studies showing that adherence decreases over time [61], [62]. Contrary to previous studies [63]–[65], we found that participants with psychological distress (depression, anxiety) had better adherence compared to those who did not have psychological distress. This uncommon association of psychological distress with better adherence merits further investigation: it is possible that efforts to sustaining ART adherence may over the long-term be source of psychological distress among some patients in the context of DRC, as a result of economic demands and/or sociocultural constraints around antiretroviral medication. Furthermore, a confounding personality trait associated with both psychological distress and ART non-adherence may also explain the observed association. Lastly, it may also be due to the nature of the K-6 scale, which was only validated in a general population in developed settings [37], [66], [67] but never tested among poor individuals with HIV in developing countries.

This study has both limitations and strengths. First we cannot assume causality of the statistically significant associations with ART adherence in this study given its cross-sectional design. It is possible that unknown or unmeasured factors could have confounded the estimates of the observed associations in our results. Second, results could be biased by socially desirable answers especially in reporting of missed medication pills in the self-reported assessment of ART adherence since the interviews were conducted by health care workers. In order to minimize this potential bias, interviewers were provided extensive training to process the survey questionnaire in a non-judgmental manner and we used a composite measure coupling both subjective (self-reported adherence) and objective (pharmacy refill adherence) measures to assess the overall adherence. On the other hand the strengths of this study include that it is rooted in the results of our qualitative study on patients on ART treatment, retreatment and lost to follow up [23]. In addition, this study derives its data from a large sample of participants selected from geographically diversified recruitment sites, including both public and private health facilities in Kinshasa, DRC. The results of this study therefore may to large extent represent the situation of patients on ART treatment in Kinshasa. However, caution is warranted in generalizing the findings of this study to a broader population. Educational attainment of our sample was higher than the general population of the DRC. Although this could reflect the general trend of HIV prevalence being higher in wealthier socio-economic groups [68], it may also be due to the fact that Kinshasa is the country’s capital and holds a more educated population.

In summary, we found that food insecurity is a significant risk factor for non-adherence to ART, and is highly prevalent among HIV-infected individuals in Kinshasa, DRC. There is urgent need of integrating effective food security strategies into HIV treatment and care programs to ensure patient adherence to ART and ultimately long-term success of HIV treatment in SSA.

## Supporting Information

Table S1 **Frequency of nonresponse: Perceptions about HIV/AIDS and ART.**
(DOC)

Table S2 **Participants’ food security status based on the HFIAS.**
(DOC)

Table S3 **Adherence status based on self-report, pharmacy refill, and combined assessment of adherence.**
(DOC)

Text S1 **Supplemental information of variables.**
(DOC)

## References

[1] Joint United Nations Programme on HIV/AIDS (2012) Global Report: UNAIDS Report on the Global AIDS Epidemic 2012 Available: http://www.unaids.org/en/media/unaids/contentassets/documents/epidemiology/2012/gr2012/20121120_UNAIDS_Global_Report_2012_with_annexes_en.pdf.Accessed 2013 Apr 25.

[2] Mills EJ, Bakanda C, Birungi J, Chan K, Ford N, et al (2011) Life expectancy of persons receiving combination antiretroviral therapy in low-income countries: a cohort analysis from Uganda. Ann Intern Med. 155: 209–216. 10.7326/0003-4819-155-4-201108160-00358 21768555

[3] Brinkhof MW, Boulle A, Weigel R, Messou E, Mathers C, et al (2009) Mortality of HIV-infected patients starting antiretroviral therapy in sub-Saharan Africa: comparison with HIV-unrelated mortality. PLoS Med 6: e1000066 10.1371/journal.pmed.1000066 19399157 PMC2667633

[4] Cohen MS, Chen YQ, McCauley M, Gamble T, Hosseinipour MC, et al (2011) Prevention of HIV-1 infection with early antiretroviral therapy. N Engl J Med 365: 493–505. 21767103 10.1056/NEJMoa1105243 PMC3200068

[5] Baeten JM, Donnell D, Ndase P, Mugo NR, Campbell JD, et al (2012) Antiretroviral prophylaxis for HIV prevention in heterosexual men and women. N Engl J Med 365: 399–410. 10.1056/NEJMoa1108524 PMC3770474 22784037

[6] Tanser F, Barnighausen T, Grapsa E, Zaidi J, Newell ML (2013) High coverage of ART associated with decline in risk of HIV acquisition in rural KwaZulu-Natal, South Africa. Science 339: 966–971. 23430656 10.1126/science.1228160 PMC4255272

[7] Sethi AK, Celentano DD, Gange SJ, Moore RD, Gallant JE (2003) Association between adherence to antiretroviral therapy and human immunodeficiency virus drug resistance. Clin Infect Dis. 37: 1112–1118. 10.1086/378301 14523777

[8] Paterson DL, Swindells S, Mohr J, Brester M, Vergis EN, et al (2000) Adherence to protease inhibitor therapy and outcomes in patients with HIV infection. Ann Intern Med 133: 21–30. 10877736 10.7326/0003-4819-133-1-200007040-00004

[9] Lima VD, Harrigan R, Bangsberg DR, Hogg RS, Gross R, et al (2009) The combined effect of modern highly active antiretroviral therapy regimens and adherence on mortality over time. J Acquir Immune Defic Syndr 50: 529–536. 19223785 10.1097/QAI.0b013e31819675e9 PMC3606956

[10] Balcha TT, Jeppsson A, Bekele A (2011) Barriers to antiretroviral treatment in Ethiopia: a qualitative study. J Int Assoc Physicians AIDS Care 10: 119–125. 10.1177/1545109710387674 21317162

[11] Curioso W, Kepka D, Cabello R, Segura P, Kurth A (2010) Understanding the facilitators and barriers of antiretroviral adherence in Peru: a qualitative study. BMC Public Health 10: 13. 20070889 10.1186/1471-2458-10-13 PMC2820472

[12] Sanjobo N, Frich JC, Fretheim A (2008) Barriers and facilitators to patients’ adherence to antiretroviral treatment in Zambia: a qualitative study. SAHARA J 5: 136–143. 18979047 10.1080/17290376.2008.9724912

[13] Tuller DM, Bangsberg DR, Senkungu J, Ware NC, Emenyonu N, et al (2009) Transportation costs impede sustained adherence and access to HAART in a clinic population in southwestern Uganda: a qualitative study. AIDS Behav 14: 778–784. 19283464 10.1007/s10461-009-9533-2 PMC2888948

[14] Mills EJ, Nachega JB, Bangsberg DR, Singh S, Rachlis B, et al (2006) Adherence to HAART: a systematic review of developed and developing nation patient-reported barriers and facilitators. PLoS Med 3(11): e438 10.1371/journal.pmed.0030438 17121449 PMC1637123

[15] Tsai AC, Bangsberg DR (2011) The importance of social ties in sustaining medication adherence in resource-limited settings. J Gen Intern Med 26: 1391–1393. 21879369 10.1007/s11606-011-1841-3 PMC3235620

[16] Ware NC, Idoko J, Kaaya S, Biraro IA, Wyatt MA, et al (2009) Explaining adherence success in sub-Saharan Africa: an ethnographic study. PLoS Med 6(1): e1000011 10.1371/journal.pmed.1000011 PMC2631046 19175285

[17] Wanyama J, Castelnuovo B, Wandera B, Mwebaze P, Kambugu A, et al (2007) Belief in divine healing can be a barrier to antiretroviral therapy adherence in Uganda. AIDS 21: 1486–1487. 17589198 10.1097/QAD.0b013e32823ecf7f

[18] Weiser SD, Fernandes KA, Anema A, Brandson EK, Lima VD, et al. (2009) Food insecurity as a barrier to antiretroviral adherence among HIV-infected individuals in British Columbia. Presented at: 5th International AIDS Society (IAS) conference on HIV pathogenesis, treatment and prevention. Cape Town, South Africa. Available: http://caps.ucsf.edu/uploads/pubs/presentations/pdf/Weiser1_IAS09.pdf. Accessed 2011 Jun 20.

[19] Weiser S, Frongillo E, Ragland K, Hogg R, Riley E, et al (2009) Food insecurity is associated with incomplete HIV RNA suppression among homeless and marginally housed HIV-infected individuals in San Francisco. J Gen intern Med 24: 14–20. 18953617 10.1007/s11606-008-0824-5 PMC2607506

[20] Kalichman S, Cherry C, Amaral C, White D, Kalichman M, et al (2010) Health and treatment implications of food insufficiency among people living with HIV/AIDS, Atlanta, Georgia. J Urban Health 87: 631–641. 20419478 10.1007/s11524-010-9446-4 PMC2900577

[21] Kalichman S, Pellowski J, Kalichman M, Cherry C, Detorio M, et al (2011) Food insufficiency and medication adherence among people living with HIV/AIDS in urban and peri-urban settings. Prevention Science 12: 324–332. 21607719 10.1007/s11121-011-0222-9 PMC7734622

[22] Peretti-Watel P, Spire B, Schiltz MA, Bouhnik AD, Heard I, et al (2006) Vulnerability, unsafe sex and non-adherence to HAART: evidence from a large sample of French HIV/AIDS outpatients. Soc Sci Med 62: 2420–2433. 16289743 10.1016/j.socscimed.2005.10.020

[23] Musumari PM, Feldman MD, Techasrivichien T, Wouters E, Ono-Kihara M, et al. (2013) “If I have nothing to eat, I get angry and push the pills bottle away from me”: A qualitative study of patient determinants of adherence to antiretroviral therapy in the Democratic Republic of Congo. AIDS Care 10.1080/09540121.2013.764391. 23383757

[24] Weiser SD, Tuller DM, Frongillo EA, Senkungu J, Mukiibi N, et al (2010) Food insecurity as a barrier to sustained antiretroviral therapy adherence in Uganda. PLoS One 5(4): e10340 10.1371/journal.pone.0010340 20442769 PMC2860981

[25] Hardon AP, Akurut D, Comoro C, Ekezie C, Irunde HF, et al (2007) Hunger, waiting time and transport costs: time to confront challenges to ART adherence in Africa. AIDS Care 19: 658–665. 17505927 10.1080/09540120701244943

[26] Weiser SD, Palar K, Frongillo EA, Tsai AC, Kumbakumba E, et al (2013) Longitudinal assessment of associations between food insecurity, antiretroviral adherence and HIV treatment outcomes in rural Uganda. AIDS 27: 000–000. 10.1097/01.aids.0000433238.93986.35 PMC4629837 23939234

[27] Wang E, McGinnis K, Fiellin D, Goulet J, Bryant K, et al (2011) Food insecurity is associated with poor virologic response among HIV-infected patients receiving antiretroviral medications. J Gen intern Med 26: 1012–1018. 21573882 10.1007/s11606-011-1723-8 PMC3157515

[28] Weiser SD, Fernandes KA, Brandson EK, Lima VD, Anema A, et al (2009) The association between food insecurity and mortality among HIV-infected individuals on HAART. J Acquir Immune Defic Syndr 52: 342–349. 19675463 10.1097/QAI.0b013e3181b627c2 PMC3740738

[29] World Bank and UNAIDS (2009) The Global Economic Crisis and HIV Prevention and Treatment Programmes: Vulnerabilities and Impact. Available: http://www.unaids.org/en/media/unaids/contentassets/dataimport/pub/report/2009/jc1734_econ_crisis_hiv_response_en.pdf. Accessed 2013 Feb 17.

[30] Food and Agriculture Organization of the United Nations (2012) The state of food insecurity in the world: Economic growth is necessary but not sufficient to accelerate reduction of hunger and malnutrition. Available: http://www.fao.org/docrep/016/i3027e/i3027e.pdf. Accessed 2013 Jan 25.

[31] Institut National de la Statistique et Fonds des Nations Unies pour l’Enfance (National Institut of Statistics and United Nations Children’s Fund) (2011) Enquête par Grappes à Indicateurs Multiples en République Démocratique du Congo 2010: Rapport Final (Democratic Republic of Congo Multiple Indicators Cluster Survey 2010: Final Report) Available: http://www.childinfo.org/files/MICS-RDC_2010_Final_Report_FR.pdf.Accessed 2012 Nov 20.

[32] Programme National Multisectoriel de lutte contre le VIH/SIDA (National Multi-sectoral Programme on HIV/AIDS) (2012) Rapport d’activité sur la riposte au VIH/SIDA en République Démocratique du Congo (Country Progress Report on HIV/AIDS Response). Available: http://www.unaids.org/en/dataanalysis/knowyourresponse/countryprogressreports/2012countries/ce_CD_Narrative_Report[1].pdf. Accessed 2012 Dec 12.

[33] Young S, Wheeler AC, McCoy SL, Weiser SD (2013) A review of the role of food insecurity in adherence to care and treatment among adult and pediatric populations living with HIV and AIDS. AIDS Behav doi:10.1007/s10461-013-0547-4. 10.1007/s10461-013-0547-4 PMC3888651 23842717

[34] Godin G, Gagne C, Naccache H (2003) Validation of a self-reported questionnaire assessing adherence to antiretroviral medication. AIDS Patient Care STDS 17: 325–332. 12952734 10.1089/108729103322231268

[35] Coates J, Swindale A, Bilinsky P (2007) *Household Food Insecurity Access scale (HFIAS) for Measurement of Food Access: Indicator guide*. Washington, DC: Food and Nutrition Technical Assistance Project, Academy for Educational Development.

[36] Kalichman SC, Simbayi LC, Cloete A, Mthembu PP, Mkhonta RN, et al (2009) Measuring AIDS stigmas in people living with HIV/AIDS: the internalized AIDS-related stigma scale. AIDS Care 21: 87–93. 19085224 10.1080/09540120802032627

[37] Kessler RC, Green JG, Gruber MJ, Sampson NA, Bromet E, et al.. (2010) Screening for serious mental illness in the general population with the K6 screening scale: results from the WHO World Mental Health (WMH) survey initiative. Int J Methods Psychiatr Res (Suppl 19): 4–22. 10.1002/mpr.310 PMC3659799 20527002

[38] Wouters E, Heunis C, van Rensburg D, Meulemans H (2008) Patient satisfaction with antiretroviral services at primary health-care facilities in the Free State, South Africa - a two-year study using four waves of cross-sectional data. BMC Health Serv Res 8: 210. 18844998 10.1186/1472-6963-8-210 PMC2575208

[39] McMahon JH, Jordan MR, Kelley K, Bertagnolio S, Hong SY, et al (2011) Pharmacy adherence measures to assess adherence to antiretroviral therapy: review of the literature and implications for treatment monitoring. Clin Infect Dis 52: 493–506. 21245156 10.1093/cid/ciq167 PMC3060901

[40] Chi BH, Cantrell RA, Zulu I, Mulenga LB, Levy JW, et al (2009) Adherence to first-line antiretroviral therapy affects non-virologic outcomes among patients on treatment for more than 12 months in Lusaka, Zambia. Int J Epidemiology 38: 746–756. 10.1093/ije/dyp004 PMC2689395 19223334

[41] Bisson GP, Gross R, Bellamy S, Chittams J, Hislop M, et al (2008) Pharmacy refill adherence compared with CD4 count changes for monitoring HIV-infected adults on antiretroviral therapy. PLoS Med 5(5): e109 10.1371/journal.pmed.0050109 18494555 PMC2386831

[42] Weidle PJ, Wamai N, Solberg P, Liechty C, Sendagala S, et al (2006) Adherence to antiretroviral therapy in a home-based AIDS care programme in rural Uganda. Lancet 368: 1587–1594. 17084759 10.1016/S0140-6736(06)69118-6

[43] Nguyen H, Zyl GV, Geboers D, Gross R, Mills EJ, et al.. (2011) Pharmacy refill data combined with self-report adherence questions improves prediction of boosted protease inhibitor regimen failure. Presented at: 6th International Conference on HIV Treatment and Prevention Adherence. Miami, USA.

[44] Goldman JD, Cantrell RA, Mulenga LB, Tambatamba BC, Reid SE, et al (2008) Simple adherence assessments to predict virologic failure among HIV-infected adults with discordant immunologic and clinical responses to antiretroviral therapy. AIDS Res Hum Retroviruses 24: 1031–1035. 18724803 10.1089/aid.2008.0035 PMC2747786

[45] Peltzer K, Ramlagan S (2011) Perceived stigma among patients receiving antiretroviral therapy: a prospective study in KwaZulu-Natal, South Africa. AIDS Care 23: 60–68. 21218277 10.1080/09540121.2010.498864

[46] Filmer D, Pritchett LH (2001) Estimating wealth effects without expenditure data–or tears: an application to educational enrollments in states of India. Demography 38(1): 115–132. 11227840 10.1353/dem.2001.0003

[47] Heaney CA, Israel BA (2008) Social networks and social support. In: Glanz K, Rimer BK, Viswanath K. Health Behavior and Health Education. San Francisco, CA: John Wiley & Sons. 189–210.

[48] Weiser SD, Tsai AC, Gupta R, Frongillo EA, Kawuma A, et al (2012) Food insecurity is associated with morbidity and patterns of healthcare utilization among HIV-infected individuals in a resource-poor setting. AIDS 26: 67–75. 21904186 10.1097/QAD.0b013e32834cad37 PMC3606954

[49] Cantrell RA, Sinkala M, Megazinni K, Lawson-Marriott S, Washington S, et al (2008) A pilot study of food supplementation to improve adherence to antiretroviral therapy among food-insecure adults in Lusaka, Zambia. J Acquir immune Defic Syndr 49: 190–195. 18769349 10.1097/QAI.0b013e31818455d2 PMC3847664

[50] Tirivayi N, Koethe JR, Groot W (2012) Clinic-based food assistance is associated with increased medication adherence among HIV-infected adults on long-term antiretroviral in Zambia. J AIDS Clin Res 3(7): 171. 23227443 10.4172/2155-6113.1000171 PMC3515057

[51] Tsai AC, Bangsberg DR, Emenyonu N, Senkungu JK, Martin JN, et al (2011) The social context of food insecurity among persons living with HIV/AIDS in rural Uganda. Soc Sci Med 73: 1717–1724. 22019367 10.1016/j.socscimed.2011.09.026 PMC3221802

[52] Tiyou A, Belachew T, Alemseged F, Biadgilign S (2012) Food insecurity and associated factors among HIV-infected individuals receiving highly active antiretroviral therapy in Jimma zone Southwest Ethiopia. Nutrition Journal 11: 51. 22824145 10.1186/1475-2891-11-51 PMC3507894

[53] Weiser SD, Hatcher A, Frongillo EA, Guzman D, Riley ED, et al (2013) Food Insecurity Is Associated with Greater Acute Care Utilization among HIV-Infected Homeless and Marginally Housed Individuals in San Francisco. J Gen Intern Med 28: 91–98. 22903407 10.1007/s11606-012-2176-4 PMC3539018

[54] Anema A, Weiser SD, Fernandes KA, Ding E, Brandson EK, et al (2011) High prevalence of food insecurity among HIV-infected individuals receiving HAART in a resource-rich setting. AIDS Care 23: 221–230. 21259135 10.1080/09540121.2010.498908

[55] World Food Programme (2003) Programming in the Era of AIDS: WFP’s Response to HIV/AIDS. Available: http://www.wfp.org/sites/default/files/Programming%20in%20the%20Era%20of%20AIDS%20WFP’s%20Response%20to%20HIV:AIDS.pdf. Accessed 2012 Nov 11.

[56] United Nations. Nutrition and HIV/AIDS. Statement by the Administrative Committee on Coordination, Sub-Committee on Nutrition as its 28th Session. Nairobi, Kenya: United Nations Administrative Committee on Coordination, Sub-Committee on Nutrition; 2001. Available: http://www.unscn.org/files/Annual_Sessions/28th_SCN_Session/28th_session_REPORT.pdf. Accessed 2012 Dec 3.

[57] Johnston BE, Ahmad K, Smith C, Rose DN (1998) Adherence to highly active antiretroviral therapy among-HIV-infected patients of the inner city. Presented at: 12th World AIDS Conference. Geneva, Switzerland.

[58] Horne R, Weinman J (1999) Patients’ beliefs about prescribed medicines and their role in adherence to treatment in chronic physical illness. Journal of Psychosom Res 47(6): 555–567. 10661603 10.1016/s0022-3999(99)00057-4

[59] Wasti SP, Simkhada P, Randall J, Freeman JV, van Teijlingen E (2012) Factors Influencing Adherence to Antiretroviral Treatment in Nepal: A Mixed-Methods Study. PLoS One 7: e35547. 22563464 10.1371/journal.pone.0035547 PMC3341373

[60] Jacquet A, Ekouevi DK, Bashi J, Aboubakrine M, Messou E, et al. (2009) Alcohol use and adherence to antiretroviral therapy in HIV-infected patients in West Africa. Presented at: 5th International AIDS Society (IAS) conference on HIV pathogenesis, treatment and prevention. Cape Town, South Africa. Available: http://www.iasociety.org/Default.aspx?pageId=11&abstractId=200721856. Accessed 2012 Jan 12.

[61] Nemes MI, Carvalho HcB, Souza MF (2004) Antiretroviral therapy adherence in Brazil. AIDS 18: S15–S20. 15322479 10.1097/00002030-200406003-00004

[62] Godin G, Cote J, Naccache H, Lambert LD, Trottier S (2005) Prediction of adherence to antiretroviral therapy: a one year longitudinal study. AIDS Care 17: 493–504. 16036235 10.1080/09540120412331291715

[63] Nel A, Kagee A (2013) The relationship between depression, anxiety and medication adherence among patients receiving antiretroviral treatment in South Africa. AIDS Care 8: 948–955. 10.1080/09540121.2012.748867 23231527

[64] Campos L, Guimaraes M, Remien R (2008) Anxiety and Depression Symptoms as risk factors for non-adherence to antiretroviral therapy in Brazil. AIDS Behav 14: 289–299. 18648925 10.1007/s10461-008-9435-8 PMC2859347

[65] Molassiotis A, Nahas-Lopez V, Chung WYR, Lam SWC, Li CKP, et al (2002) Factors associated with adherence to antiretroviral medication in HIV-infected patients. Int J STD AIDS 13: 301–310. 11972933 10.1258/0956462021925117

[66] Furukawa TA, Kessler RC, Slade T, Andrews G (2003) The performance of the K6 and K10 screening scales for psychological distress in the Australian National Survey of Mental Health and Well-Being. Psychol Med 33: 357–362. 12622315 10.1017/s0033291702006700

[67] Andersen LS, Grimsrud A, Myer L, Williams DR, Stein DJ, et al (2011) The psychometric properties of the K10 and K6 scales in screening for mood and anxiety disorders in the South African Stress and Health study. Int J Methods Res 20: 215–223. 10.1002/mpr.351 PMC6878543 22113964

[68] Joint United Nations Programme on HIV/AIDS & World Health Organization (2009) AIDS Epidemic update. Available: http://data.unaids.org/pub/Report/2009/JC1700_Epi_Update_2009_en.pdf. Accessed 2012 Mar 23.

