# Supplementary material for: Food Insecurity Is Associated with Increased Risk of Non-Adherence to Antiretroviral Therapy among HIV-Infected Adults in the Democratic Republic of Congo: A Cross-Sectional Study
Source: PLoS One. 2014 Jan 15;9(1):e85327. doi: 10.1371/journal.pone.0085327 (PMC3893174; doi:10.1371/journal.pone.0085327)
Supplement: Table S1 — Frequency of nonresponse: Perceptions about HIV/AIDS and ART. (DOC) [file pone.0085327.s001.doc]

| **Table S1**. Frequency of nonresponse: Perceptions about HIV/AIDS and ART | | |
| --- | --- | --- |
|  | n | % |
| **Sociocultural/Religious beliefs** |  |  |
| God/prayers can cure HIV |  |  |
| Nonresponse | 43 | 4.8 |
| Traditional healers/medicine can cure HIV |  |  |
| Nonresponse | 42 | 4.7 |
| ART is effective when associated with prayers | |  |
| Nonresponse | 52 | 5.8 |
| **Perceptions about food and ART** |  |  |
| ART not necessary without food |  |  |
| Nonresponse | 29 | 3.2 |
| ART not effective without food |  |  |
| Nonresponse | 37 | 4.1 |
| ART can be harmful without food |  |  |
| Nonresponse | 28 | 3.1 |
| **Perceptions about ART adherence** |  |  |
| Short treatment interruption is not harmful for a long term ART user |  |  |
| Nonresponse | 19 | 2.1 |
| Skipping few ART doses is not harmful for a long-term ART user |  |  |
| Nonresponse | 12 | 1.3 |
| Skipping ART doses can worsen the disease |  |  |
| Nonresponse | 13 | 1.4 |
| ART should be taken life-long |  |  |
| Nonresponse | 10 | 1.1 |
| **Perception about ART** |  |  |
| Perceived effectiveness of ART |  |  |
| Nonresponse | 0 | 0 |
| Perceived ART harmfulness |  |  |
| Nonresponse | 0 | 0 |
