# Supplementary material for: Food Insecurity Is Associated with Increased Risk of Non-Adherence to Antiretroviral Therapy among HIV-Infected Adults in the Democratic Republic of Congo: A Cross-Sectional Study
Source: PLoS One. 2014 Jan 15;9(1):e85327. doi: 10.1371/journal.pone.0085327 (PMC3893174; doi:10.1371/journal.pone.0085327)
Supplement: Table S2 — Participants’ food security status based on the HFIAS. (DOC) [file pone.0085327.s002.doc]

| **Table S2**. Participants food security status based on the HFIAS | | | |
| --- | --- | --- | --- |
|  |  | n | % |
| 1 | Food secure | 386 | 43.0 |
| 2 | Mildly food insecure | 9 | 1.0 |
| 3 | Moderately food insecure | 46 | 5.1 |
| 4 | Severely food insecure | 457 | 50.9 |
| HFIAS, Household food Insecurity Access Scale | | | |
