# Supplementary material for: Food Insecurity Is Associated with Increased Risk of Non-Adherence to Antiretroviral Therapy among HIV-Infected Adults in the Democratic Republic of Congo: A Cross-Sectional Study
Source: PLoS One. 2014 Jan 15;9(1):e85327. doi: 10.1371/journal.pone.0085327 (PMC3893174; doi:10.1371/journal.pone.0085327)
Supplement: Table S3 — Adherence status based on self-report, pharmacy refill, and combined assessment of adherence. (DOC) [file pone.0085327.s003.doc]

| **Table S3**. Adherence status based on self-report, pharmacy refill, and combined assessment of adherence | | | | |
| --- | --- | --- | --- | --- |
|  | Non-Adherent |  | Adherent |  |
|  | n | % | n | % |
| Self-reported adherence | 87 | 9.7 | 811 | 90.3 |
| Pharmacy refill adherence | 154 | 17.1 | 744 | 82.1 |
| Combined adherence | 188 | 20.9 | 710 | 79.1 |
