# Supplementary material for: Food Insecurity Is Associated with Increased Risk of Non-Adherence to Antiretroviral Therapy among HIV-Infected Adults in the Democratic Republic of Congo: A Cross-Sectional Study
Source: PLoS One. 2014 Jan 15;9(1):e85327. doi: 10.1371/journal.pone.0085327 (PMC3893174; doi:10.1371/journal.pone.0085327)
Supplement: Text S1 — Supplemental information of variables. (DOC) [file pone.0085327.s004.doc]

**Supplemental information of variables**

*HIV/AIDS knowledge*

HIV/AIDS knowledge was assessed with 8 items which explored 1) knowledge about prevention on two main routes of HIV transmission (consistent condom use can prevent HIV transmission; HIV can be transmitted through breast feeding), 2) misconception about HIV/AIDS (HIV can be transmitted through sharing food; mosquito bites; supernatural means), and 3) knowledge on HIV/AIDS disease progression (a healthy looking person may have HIV/AIDS; a positive test means that one is infected or life; a HIV infected person may not have HIV/AIDS symptoms). We created a score by giving 1 for correct answer and 0 for incorrect or don’t know answers. Scores from 0 to 4 were considered poor knowledge and from 5 to 8 were considered good knowledge.

*Perceived Social Support*:

We developed questions to capture key dimensions of social support including emotional, tangible and informational support [1]. Participants were asked whether or not they received emotional, financial, nutritional, physical care assistance, and whether such supports were obtained from family or non-family members.

*Perceived satisfaction with quality of care*

To measure participant satisfaction with care, we adapted 6 items previously used elsewhere [2]. Participants were asked on a 5-point likert scale (ranging from very unsatisfied to very satisfied) how satisfied they were with 1) medical care at the health facility 2) privacy during examinations 3) respect shown by health care providers 4) information provided by health care providers about antiretroviral therapy 5) facility opening hours 6) waiting time before consultation. Very unsatisfied, unsatisfied, and neutral were converted to 0 and satisfied and very satisfied were converted to one. Scores ranged from 0 to 6 with higher scores indicating higher greater degree of satisfaction.

*Wealth index*

We constructed a household wealth index based on the method proposed by Filmer and Pritchett, and which relies on the principal component analysis [3]. The household assets and characteristics used to derive the wealth index included electricity, running water, television, radio, refrigerator, mobile phone, telephone, stove or cooker, chair(s), bed, bicycle, motorcycle, car(s), number of rooms per effective household size. The index was used as a continuous variable, with higher values indicating greater household wealth.

*Perceptions about HIV/AIDS and ART*

We devised 12 items to assess participants’ perceptions about HIV/AIDS and ART based on results from our qualitative study [4]. Items specifically addressed sociocultural/religious beliefs (1. God/prayers can cure HIV, 2. Traditional healers/medicines can cure HIV, and 3. ART works better when combined with prayers), perceptions about ART and food (1. ART is not necessary, 2. ART is not effective, and 3. ART can be harmful, when taken without food), perception about ART adherence (1. Short ART interruption is not harmful for a long-term ART user, 2. skipping few ART doses is not harmful for a long-term ART user, 3. Skipping ART doses can worsen the disease, and 4. ART should be taken life-long), perceived effectiveness, and perceived harmfulness of ART. Participants were asked if they agreed or disagree with, or did not know, the above statements, at the exceptions of the last two items which were yes-or-no questions.

References

1. Heaney CA, Israel BA (2008) Social networks and social support. In: Glanz K, Rimer BK, Viswanath K. Health Behavior and Health Education. San Francisco, CA: John Wiley & Sons. pp.189-210.
2. Wouters E, Heunis C, van Rensburg D, Meulemans H. (2008) Patient satisfaction with antiretroviral services at primary health-care facilities in the Free State, South Africa - a two-year study using four waves of cross-sectional data. Bmc Health Services Research 8:210.
3. Filmer D, Pritchett LH. (2001) Estimating wealth effects without expenditure data—or tears: an application to educational enrollments in states of India. Demography 38(1): 115-132
4. Musumari PM, Feldman MD, Techasrivichien T, Wouters E, Ono-Kihara M, et al. (2013) "If I have nothing to eat, I get angry and push the pills bottle away from me": A qualitative study of patient determinants of adherence to antiretroviral therapy in the Democratic Republic of Congo. AIDS Care http://dx.doi.org/10.1080/09540121.2013.764391.
